# Association between admission inflammatory indicators and 3-year mortality risk in geriatric patients after hip fracture surgery: a retrospective analysis of a prospective cohort study

**DOI:** 10.3389/fsurg.2024.1440990

**Published:** 2024-08-20

**Authors:** Yimin Chen, Chao Tu, Gang Liu, Weidong Peng, Jing Zhang, Yufeng Ge, Zhelun Tan, Mingjian Bei, Feng Gao, Maoyi Tian, Minghui Yang, Xinbao Wu

**Affiliations:** ^1^ Peking University Fourth School of Clinical Medicine, Beijing, China; ^2^ Department of Orthopaedics and Traumatology, Beijing Jishuitan Hospital, Capital Medical University, Beijing, China; ^3^ National Center for Orthopaedics, Beijing, China; ^4^ School of Population Health, University of New South Wales, Sydney, NSW, Australia; ^5^ School of Public Health, Harbin Medical University, Harbin, Heilongjiang, China; ^6^ The George Institute for Global Health, University of New South Wales, Sydney, NSW, Australia

**Keywords:** geriatric, hip fracture, MLR, NLR, PLR, SII, CRP, mortality

## Abstract

**Background:**

Recent research indicates that the monocyte lymphocyte ratio (MLR), neutrophil lymphocyte ratio (NLR), platelet lymphocyte ratio (PLR), C-reactive protein (CRP), and systemic immune-inflammation index (SII) may serve as valuable predictors of early postoperative mortality in elderly individuals with hip fractures. The primary objective of the study was to examine the association between preoperative MLR, NLR, PLR, CRP, and SII levels and 3-year mortality risk in geriatric patients after hip fracture surgery.

**Patients and methods:**

The study included patients aged 65 years or older who underwent hip fracture surgery between November 2018 and November 2019. Admission levels of MLR, NLR, PLR, CRP, and SII were measured. The median follow-up period was 3.1 years. Cox proportional hazards models were used to calculate the hazard ratio (HR) for mortality with adjusting for potential covariates. Time-dependent receiver operating characteristic (ROC) curves were employed to assess the predictive capability of inflammatory indicators for mortality.

**Results:**

A total of 760 patients completed the follow-up (79.4 ± 7.8 years, 71.1% female). A higher preoperative MLR was found to be significantly associated with an increased 3-year postoperative mortality risk (HR 1.811, 95% CI 1.047–3.132, *P* = 0.034). However, no significant correlations were observed between preoperative NLR, PLR, CRP, SII and 3-year mortality. The areas under the ROC curve (AUCs) of MLR for predicting 30-day, 120-day, 1-year, and 3-year mortality were 0.74 (95% CI 0.53–0.95), 0.70 (95% CI 0.57–0.83), 0.67 (95% CI 0.60–0.74), and 0.61 (95% CI 0.56–0.66), respectively.

**Conclusion:**

Preoperative MLR is a useful inflammatory marker for predicting 3-year mortality in elderly hip fracture patients, but its predictive ability diminishes over time.

## 1 Introduction

Hip fracture is a major health concern for elderly individuals due to its high morbidity and mortality (1). The one-year mortality rate in geriatric patients with hip fracture was reported as high as 12%–37%, and mortality risk may persist over five years (2, 3). Therefore, identifying predictive factors of postoperative mortality is crucial for surgeons to identify high-risk patients and plan interventions for improvement.

Recent studies have shown that preoperative immune-inflammation indicators, including lymphocyte ratio (NLR), platelet lymphocyte ratio (PLR), monocyte lymphocyte ratio (MLR), C-reactive protein (CRP), and systemic immune-inflammation index (SII), are correlated with increased all-cause mortality in elderly patients after surgeries (4–11). Recent studies have found that NLR, MLR, and CRP levels could predict mortality in elderly patients after hip fracture surgery (7, 9), but there is some controversy surrounding their association with postoperative mortality (12, 13).

Previous studies have primarily looked at the relationship between inflammation indicators and short-term mortality in geriatric hip fracture patients. This study aims to explore the connection between preoperative inflammatory indicators and long-term (3-year) all-cause mortality, as well as how these indicators predict mortality over time.

## 2 Materials and methods

### 2.1 Study design

The study took place at a hospital with a co-managed orthogeriatric hip fracture care pathway. Prior to commencing the study, ethical approval was obtained from the Institutional Review Board at Peking University Health Science Centre (IRB00001052-17021) and Biomedical Ethics Committee at Beijing Jishuitan Hospital (201807-11). All participants provided written informed consent before data collection. For illiterate participants, informed consent to participate was obtained from their literate legal guardians. This retrospective study used previously collected baseline data from our observational study on elderly hip fracture patients in China (Clinical Trials.gov Identifier: NCT03184896) (14).

We initially enrolled 1,092 patients aged 65 and older who underwent surgery for hip fracture within three weeks of injury between November 2018 and November 2019, following a described clinical method (14). Among these patients, there were 556 (50.9%) femoral neck fractures (FNFs), 511 (46.8%) intertrochanteric fractures (ITFs), and 25 (2.3%) subtrochanteric fractures (STFs). The primary cause of hip fractures was low-energy falls (87.1%). For FNFs, there were 112 (20.1%) undisplaced fractures (Garden I/II) and 444 (79.9%) displaced fractures (Garden III/IV). In ITFs, there were 132 (25.8%) 31A1 type, 348 (68.1%) 31A2 type, and 31 (6.1%) 31A3 type according to the Arbeitsgemeinschaft für Osteosynthesefragen/Orthopaedic Trauma Association (AO/OTA) classification system.

During the screening process, patients were excluded based on the following criteria: (1) incomplete baseline or laboratory data; (2) presence of acute or chronic infection in other tissues or organs prior to laboratory testing; (3) pathological or periprosthetic fractures; (4) terminal malignancies. Patients were followed up by phone for a median duration of 3.1 years post-surgery.

### 2.2 Inflammatory indicators calculation

In the present investigation, admission laboratory test data from the emergency department (ED) was gathered in order to calculate inflammatory indicators including MLR, NLR, PLR, and SII. The calculation of these indicators was conducted as follows: (1) MLR = Monocyte count (×10^9^/L)/Lymphocyte count (×10^9^/L); (2) NLR = Neutrophil count (×10^9^/L)/Lymphocyte count (×10^9^/L); (3) PLR = Platelet count (×10^9^/L)/Lymphocyte count (×10^9^/L); (4) SII = [Platelet count (×10^9^/L) × Neutrophil count (×10^9^/L)]/Lymphocyte count (×10^9^/L).

### 2.3 Data collection and outcome measurements

The study collected demographic and perioperative information, including age, sex, weight, height, BMI, smoking and drinking habits, education level, living situation, and baseline medical conditions such as hypertension, diabetes, cognitive and visual impairments. Education level was categorized into five levels from illiterate to university or higher. Cognitive function was evaluated with the MMSE-China (15), and those with a score of 23 or lower were classified as having cognitive impairment. The Charlson Comorbidity Index (CCI) was used to assess overall medical condition. Perioperative factors included time from injury to ED arrival, fracture type, American Society of Anesthesiologists (ASA) scores, anaesthesia type, operation type, and length of stay (LOS). Serum albumin (Alb) level at admission was used to indicate nutritional status. Left ventricular ejection fraction (LVEF) from echocardiogram conducted at ED was used to evaluate cardiac function. Undisplaced FNFs were treated with cannulated screw fixation (CS), while displaced fractures were treated with hip arthroplasty. Stable ITFs (31A1) were treated with either dynamic hip screw (DHS), plate and screws, or cephalomedullary devices, while unstable ITFs (31A2 and 31A3) and STFs were treated mainly with cephalomedullary devices. In this study, operations were categorized as internal fixation (CS, DHS, locking plate, and intramedullary nailing) or arthroplasty.

All patients underwent a three-year post-surgery follow-up via telephone with orthopaedists. The aim of this study was to assess all-cause mortality rates during the 3-year period.

### 2.4 Statistical analysis

Parametric data were shown with means and standard deviations (SD), while non-parametric data were presented with medians and interquartile ranges (IQR). Categorical data were shown using frequencies and numerical distributions. The Chi-squared test was used to compare categorical variables, while Student's *t*-test or Mann-Whitney *U*-test were used for continuous variables based on their distribution.

In the present study, covariates were determined based on baseline variables that were deemed clinically significant or showed a univariate correlation with the outcome. Multivariate Cox proportional hazards models were utilized to assess the association between inflammatory indicators and the postoperative mortality risk, adjusting for covariates. Additionally, restricted cubic splines (RCS) with three knots at the 10th, 50th, and 90th percentiles were used to flexibly model the correlation of these indicators with 3-year mortality after adjusting for covariates. The reference value (HR = 1) was established at the 50th percentile for each indicator. Time-dependent receiver operating characteristic (ROC) curves were generated to evaluate the predictive ability of inflammatory indicators for mortality. The optimal cutoff value of indicator for predicting 3-year mortality was calculated by Youden index.

The analyses were conducted using the statistical software packages R 4.1.1 (http://www.R-project.org, The R Foundation). A two-tailed test was used with a significance level of *P* < 0.05.

## 3 Results

**Figure 1:**
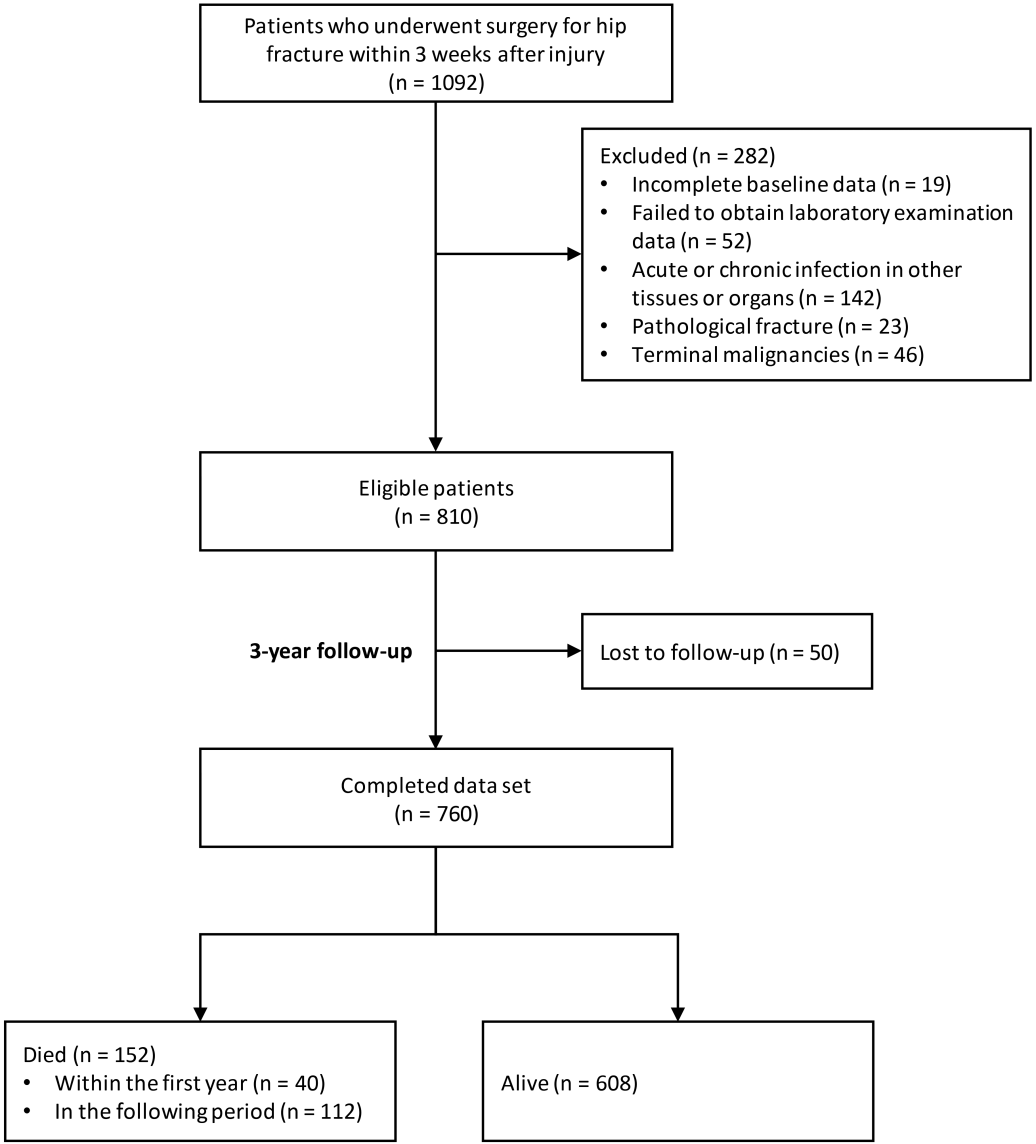
Flowchart of the study.

Figure 1 displays the flowchart of the study, indicating that 810 patients met the eligibility criteria following exclusion. The follow-up duration for these patients was recorded as 3.1 (2.8, 3.4) years. Our final analysis included 760 patients, with 50 lost to follow-up. Of these patients, 152 (20.0%) deceased, with 40 (5.3%) deaths occurring within the first year.

Table 1 presents the baseline characteristics of the entire patient cohort. The average age was 79.4 years, with 71.1% female and 49.2% ITFs. The median time from injury to arrival at the ED was 13.7 h, and the median time from admission to surgery was around 26.6 h. The median LOS was approximately 5 days. Table 1 shows that surviving patients exhibited statistically significantly lower levels of inflammatory indicators, including CRP, MLR, PLR, and SII, compared to deceased patients (*P* = 0.001; *P* < 0.001; *P* = 0.004; *P* = 0.045, respectively). Additionally, deceased patients displayed lower levels of BMI, LVEF, albumin, and hemoglobin (22.0 ± 4.1 vs. 23.2 ± 3.7; 64.3 ± 4.9 vs. 65.7 ± 4.1; 38.0 ± 4.0 vs. 41.1 ± 3.1; 107.7 ± 17.4 vs. 117.0 ± 17.6, respectively). Furthermore, a higher prevalence of cognitive impairment was observed in the deceased group [24 (15.8%) vs. 51 (8.4%)].

**Table 1:** Baseline characteristics.

Variables	Total (*n* = 760)	Alive (*n* = 608)	Died (*n* = 152)	*P*-value
Age, years, mean ± SD	79.4 ± 7.8	78.3 ± 7.7	83.8 ± 6.7	<0.001
Female, *n* (%)	540 (71.1)	443 (72.9)	97 (63.8)	0.028
BMI, kg/m^2^, mean ± SD	23.0 ± 3.8	23.2 ± 3.7	22.0 ± 4.1	<0.001
Comorbidities, *n* (%)				
Diabetes	219 (28.8)	183 (30.1)	36 (23.7)	0.118
Hypertension	492 (64.7)	392 (64.5)	100 (65.8)	0.761
CAD	183 (24.1)	132 (21.7)	51 (33.6)	0.002
Cognitive impairment	75 (9.9)	51 (8.4)	24 (15.8)	0.006
Visual impairment	316 (41.6)	251 (41.3)	65 (42.8)	0.740
CCI, *n* (%)				0.002
0	260 (34.2)	224 (36.8)	36 (23.7)	
1	272 (35.8)	215 (35.4)	57 (37.5)	
2	149 (19.6)	116 (19.1)	33 (21.7)	
≥3	79 (10.4)	53 (8.7)	26 (17.1)	
Ever or current smokers, *n* (%)	127 (16.7)	94 (15.5)	33 (21.7)	0.065
Current drinkers, *n* (%)	38 (5.0)	28 (4.6)	10 (6.6)	0.318
Educational level, *n* (%)				0.240
Illiterate	140 (18.4)	106 (17.4)	34 (22.4)	
Primary school or lower	190 (25.0)	147 (24.2)	43 (28.3)	
High school	210 (27.6)	174 (28.6)	36 (23.7)	
University or higher	220 (28.9)	181 (29.8)	39 (25.7)	
MMSE scale, mean ± SD	20.6 ± 5.2	21.0 ± 4.8	18.7 ± 6.1	<0.001
Live alone, *n* (%)	82 (10.8)	69 (11.3)	13 (8.6)	0.320
Live at home, *n* (%)	456 (60.0)	367 (60.4)	89 (58.6)	0.684
Non-ground level fall, *n* (%)	82 (10.9)	68 (11.3)	14 (9.3)	0.483
Time from injury to emergence arrival, hour, median (IQR)	13.7 (4.4, 40.8)	12.6 (4.4, 40.8)	19.9 (4.3, 44.8)	0.188
Fracture type, *n* (%)				0.089
FNF	370 (48.7)	305 (50.2)	65 (42.8)	
ITF	374 (49.2)	288 (47.4)	86 (56.6)	
STF	16 (2.1)	15 (2.5)	1 (0.7)	
Fracture side, *n* (%)				0.678
Left	373 (49.1)	297 (48.8)	76 (50)	
Right	376 (49.5)	303 (49.8)	73 (48)	
Bilateral	11 (1.4)	8 (1.3)	3 (2)	
LVEF, %, mean ± SD	65.4 ± 4.3	65.7 ± 4.1	64.3 ± 4.9	<0.001
ASA score, *n* (%)				<0.001
I	96 (12.6)	87 (14.3)	9 (5.9)	
II	381 (50.1)	323 (53.1)	58 (38.2)	
≥III	283 (37.2)	198 (32.6)	85 (55.9)	
Anaesthesia type, *n* (%)				1
Spinal	758 (99.7)	606 (99.7)	152 (100)	
General	2 (0.3)	2 (0.3)	0 (0)	
TTS, hours, median (IQR)	26.6 (8.4, 47.2)	26.6 (8.4, 47.1)	26.7 (8.4, 47.7)	0.658
Operation type, *n* (%)				0.738
Internal fixation	466 (61.3)	371 (61)	95 (62.5)	
Arthroplasty	294 (38.7)	237 (39)	57 (37.5)	
LOS, days, median (IQR)	5.0 (4.0, 6.4)	4.9 (3.9, 6.2)	5.5 (4.5, 6.8)	0.005
Laboratory test at admission				
ALT, IU/L, median (IQR)	15.0 (11.0, 20.0)	15.0 (12.0, 19.2)	14.0 (10.8, 20.0)	0.130
AST, IU/L, median (IQR)	18.5 (16.0, 23.0)	18.0 (16.0, 23.0)	19.0 (16.0, 24.2)	0.103
ALP, IU/L, median (IQR)	63.0 (52.0, 80.0)	63.0 (52.0, 80.0)	65.0 (53.0, 84.0)	0.407
Albumin, g/L, mean ± SD	40.5 ± 3.5	41.1 ± 3.1	38.0 ± 4.0	<0.001
Crea, umol/L, mean ± SD	61.0 (50.0, 79.0)	59.0 (48.0, 75.0)	68.0 (57.0, 91.0)	<0.001
Hb, g/L, mean ± SD	115.2 ± 18.0	117.0 ± 17.6	107.7 ± 17.4	<0.001
Inflammatory indicators				
WBC, × 10^9^/L, mean ± SD	9.1 ± 2.8	9.1 ± 2.7	8.9 ± 3.0	0.403
CRP, mg/L, median (IQR)	9.0 (3.0, 31.2)	8.0 (3.0, 28.0)	14.5 (3.8, 51.2)	0.001
MLR, median (IQR)	0.5 (0.4, 0.8)	0.5 (0.4, 0.7)	0.6 (0.4, 0.8)	<0.001
NLR, median (IQR)	5.9 (4.1, 9.1)	5.8 (4.1, 9.1)	6.5 (4.8, 9.3)	0.068
PLR, median (IQR)	171.3 (124.5, 235.9)	167.0 (124.2, 225.2)	193.2 (132.1, 285.6)	0.004
SII, median (IQR)	1,126.8 (762.5, 1,819.7)	1,095.0 (757.5, 1,781.0)	1,285.5 (786.0, 2,051.4)	0.045

SD, standard deviation; BMI, body mass index; CAD, coronary artery disease; CCI, Charlson Comorbidity Index; MMSE, mini-mental state examination; FNF, femoral neck fracture; ITF, intertrochanteric fracture; STF, subtrochanteric fracture; LVEF, left ventricular ejection fraction; ASA, American Society of Anesthesiologists; TTS, time to surgery; LOS, length of stay; IQR, interquartile range; ALT, Alanine aminotransferase; AST, Aspartate aminotransferase; ALP, alkaline phosphatase; Crea, (serum) creatinine; Hb, hemoglobin; WBC, white blood cell; CRP, C-reactive protein; MLR, monocyte lymphocyte ratio; NLR, neutrophil lymphocyte ratio; PLR, platelet lymphocyte ratio; SII, systemic immune-inflammation index.

**Figure 2:**
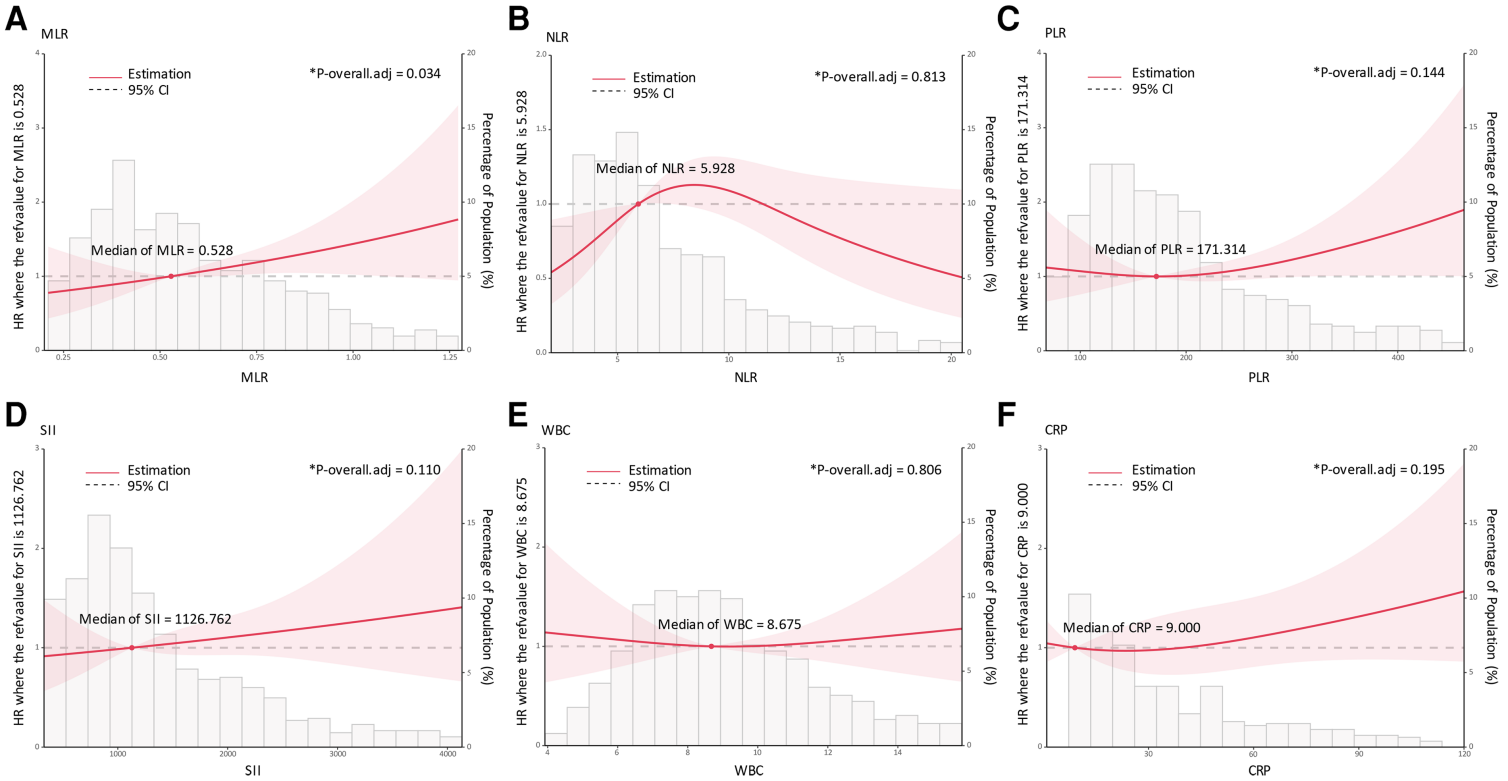
Association of admission inflammatory indicators with 3-year mortality risk using restricted cubic spline regression models. **(A)**, MLR; **(B)**, NLR; **(C)**, PLR; **(D)**, SII; **(E)**, WBC; **(F)**, CRP; *Died vs. Alive (152 vs. 608), adjusted for age, sex, BMI, CAD, cognitive impairment, CCI, LVEF, albumin, Crea, and Hb. HR, hazard ratio; MLR, monocyte lymphocyte ratio, NLR, neutrophil lymphocyte ratio; PLR, platelet lymphocyte ratio, SII, systemic immune-inflammation index; WBC, white blood cell; CRP, C-reactive protein; BMI, body mass index; CAD, coronary artery disease; CCI, Charlson Comorbidity Index; LVEF, left ventricular ejection fraction; Crea, creatinine; Hb, hemoglobin.

In an unadjusted model, the MLR, PLR, and CRP demonstrated significant associations with the 3-year mortality risk, as shown in Table 2. Upon controlling for age, sex, and BMI in Model 1, MLR (HR 2.105, 95% CI 1.258–3.523), PLR (HR 1.002, 95% CI 1.001–1.003), and CRP (HR 1.009, 95% CI 1.005–1.014) maintained statistically significant correlations with mortality risk. However, upon further adjustment for all covariates in Model 2, it was observed that only MLR remained significantly associated with postoperative mortality (HR 1.811, 95% CI 1.047–3.132) (Figure 2).

**Table 2:** Hrs of inflammatory indicators for 3-year mortality.

Indicators	Died vs. alive (152 vs. 608)					
	Unadjusted		Model 1^a^		Model 2^b^	
	HR (95% CI)	*P* value	HR (95% CI)	*P* value	HR (95% CI)	*P* value
MLR	3.079 (1.906–4.974)	<0.001	2.105 (1.258–3.523)	0.005	1.811 (1.047–3.132)	0.034
NLR	1.019 (0.988–1.051)	0.241	1.002 (0.969–1.035)	0.910	1.004 (0.971–1.038)	0.813
PLR	1.002 (1.001–1.004)	<0.001	1.002 (1.001–1.003)	0.006	1.001 (1.000–1.003)	0.144
SII	1.000 (0.999–1.000)	0.073	1.000 (0.999–1.000)	0.097	1.000 (0.999–1.000)	0.110
WBC	0.978 (0.921–1.038)	0.457	0.980 (0.922–1.042)	0.523	1.008 (0.949–1.070)	0.806
CRP	1.010 (1.005–1.014)	<0.001	1.009 (1.005–1.014)	<0.001	1.003 (0.998–1.008)	0.195

HR, hazard ratio; CI, confidential interval; MLR, monocyte lymphocyte ratio; NLR, neutrophil lymphocyte ratio; PLR, platelet lymphocyte ratio; SII, systemic immune-inflammation index; WBC, white blood cell; CRP, C-reactive protein; BMI, body mass index; CAD, coronary artery disease; CCI, Charlson Comorbidity Index; LVEF, left ventricular ejection fraction; Crea, (serum) creatinine; Hb, hemoglobin.

^a^ Model 1, adjusted for age, sex, and BMI.

^b^ Model 2, adjusted for age, sex, BMI, CAD, cognitive impairment, CCI, LVEF, albumin, Crea, and Hb.

**Figure 3:**
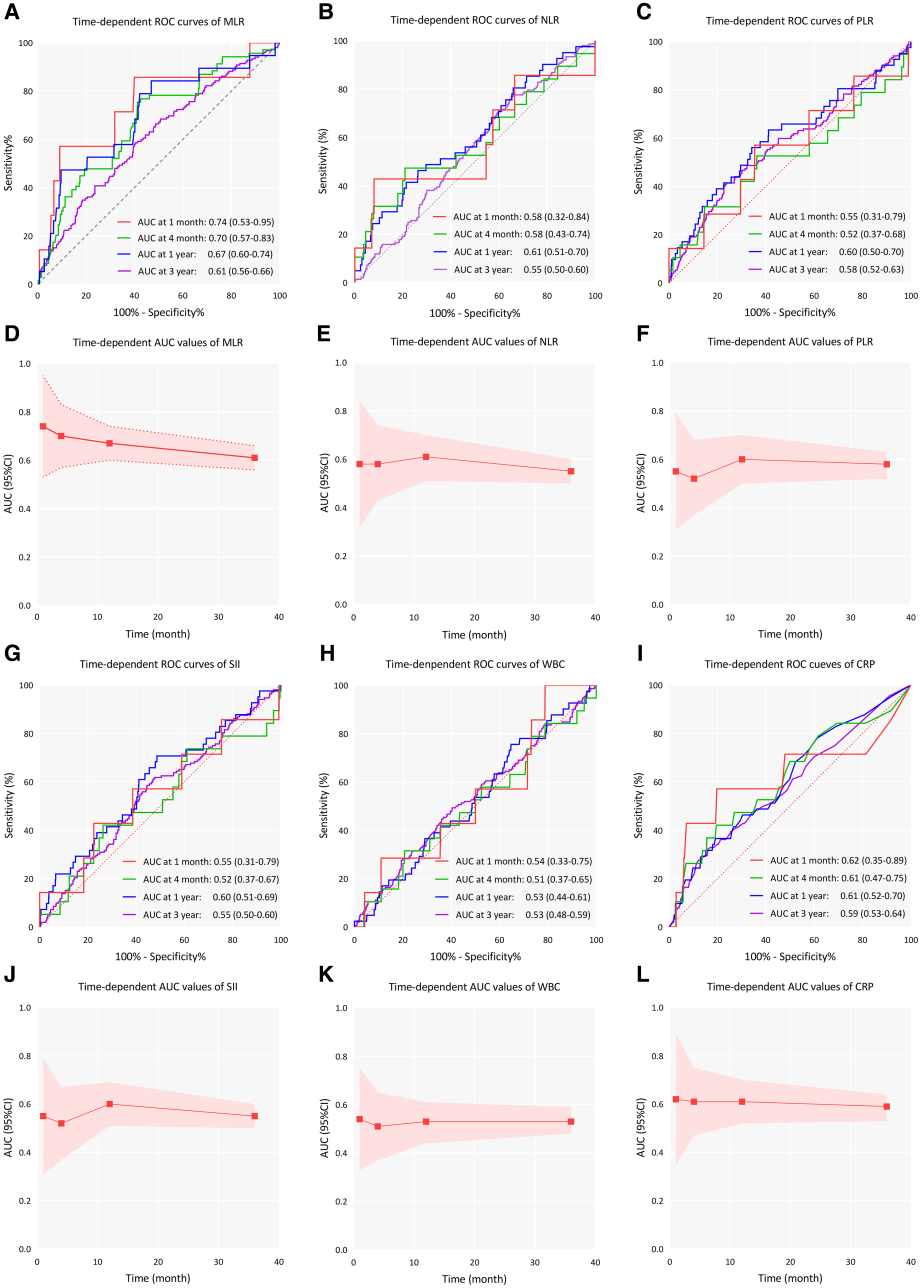
Time-dependent ROC curves and time-dependent AUC values (with 95% CI band) of inflammatory indicators for predicting all-cause mortality. **(A–C)**, Time-dependent ROC curves of MLR, NLR, and PLR; **(D–F)**, Time-dependent AUC values of MLR, NLR, and PLR; **(G–I)**, Time-dependent ROC curves of SII, WBC, and CRP; **(J–L)**, Time-dependent AUC values of SII, WBC, and CRP; AUC, area under curve; ROC, receiver operating characteristic curve; CI, confidence interval; MLR, monocyte lymphocyte ratio, NLR, neutrophil lymphocyte ratio; PLR, platelet lymphocyte ratio, SII, systemic immune-inflammation index; WBC, white blood cell; CRP, C-reactive protein.

The time-dependent ROC analysis revealed a decline in the predictive performance of MLR based on the areas under the ROC curve (AUCs) for mortality at 30 days, 120 days, 1 year, and 3 years (Figure 3). Specifically, the AUC values decreased over time, with corresponding values of 0.74 (95% CI 0.53–0.95), 0.70 (95% CI 0.57–0.83), 0.67 (95% CI 0.60–0.74), and 0.61 (95% CI 0.56–0.66), respectively. Other inflammatory indicators including NLR, PLR, SII, WBC, and CRP exhibited a consistent pattern of declining AUC values over time.

Lastly, the normal range for MLR was established between the 2.5% and 97.5% percentiles (0.210–1.225). The optimal cutoff value of MLR for predicting the 3-year mortality risk was set as 0.581 (sensitivity 0.586, specificity 0.597).

## 4 Discussion

This study found that MLR is one of the risk factors for death within three years after surgery in elderly patients with hip fractures. There was a positive correlation between the MLR level and the risk of mortality. However, other inflammatory indicators including NLR, PLR, SII and CRP were not found to be significant in predicting prognosis. Our study identified the significance of MLR as a predictor of long-term mortality in geriatric patients after hip fracture surgery.

The incidence of hip fracture is often high in elderly patients, with a high mortality and disability rate, which brings a huge economic burden to the society (16). A hip fracture is a traumatic strike that might cause systemic inflammatory response. Changes in these immune markers help predict the complications and death in patients (17). Lymphocyte, neutrophil, platelet, CRP are commonly used in the laboratory and are easy to obtain and measure. These indicators can reflect the systemic immune response, and the predictive value of MLR and NLR indicators in various tumors has been proven (5, 18–20). Some studies have focused on the relationship between changes in inflammatory markers at the early stage of hip fracture and patient mortality, but there is still no definitive conclusion. The underlying reasons for the association between mortality after hip fractures and immune markers, while not yet clear, are likely related to the inflammatory response, comorbidities, and age-related changes in immune function induced by fractures. Martin and colleagues discovered that inflammatory markers such as WBC count, NLR, and CRP in the acute phase of trauma were not indicative of long-term prognosis. However, alterations in these markers three weeks post-surgery may hold significance (21). Sun et al. discovered that the inflammatory markers TNF-a, IL-6, and IL-10 were significant prognostic indicators for postoperative complications and mortality within a 1-year period among elderly individuals with hip fractures (22). However, there is still a lack of research on the impact of these inflammatory indicators on the long-term mortality of patients.

This study found the significance of MLR in predicting postoperative mortality in elderly patients with hip fractures. Analysis of the ROC curve revealed that MLR was able to accurately predict 30-day, 120day, 1-year, and 3-year mortality outcomes, although its predictive value diminished with increasing time elapsed. The study conducted by Tekin et al. utilized MLR as a metric and identified a noteworthy association between the 1-year postoperative mortality rate of elderly individuals with hip fractures. The researchers determined that a cutoff value of 0.54 and an AUC of 0.847 were indicative of a strong predictive capability (23). Similarly, Bingol et al. conducted a study on the predictive significance of MLR one month and one year after surgery, and obtained cut-off values of 0.65 (AUC value 0.849) and 0.635 (AUC value 0.843), respectively (9). These studies confirmed that MLR has a predictive value for postoperative death in elderly patients with hip fracture, which is similar to the results of our study. In both studies, NLR was determined to be significantly correlated with 1-year postoperative mortality, whereas in the present study, no definitive association was observed with long-term mortality at 3 years post-surgery. In a study examining long-term postoperative mortality in elderly patients who had suffered hip fractures, NLR was significantly correlated with 4-year mortality, but not associated with 1-month or 1-year mortality (24). We considered that the reason for the different results might be related to the fact that the patients included in this study were all under the co-management mode, and the mortality rate of the patients in this study was significantly lower than that in those two studies. Even with such a low mortality rate, the prediction value by MLR is still significant, which further reflects the value of MLR as an observational indicator in predicting the risk of mortality.

In this study, NLR, PLR, and SII were not significant in predicting long-term mortality, indicating MLR may be a relatively more effective indicator. Neutrophils serve as the primary effector cells of the innate immune response, while lymphocytes play a regulatory role in the immune system (25, 26). NLR is a measure of inflammation intensity based on the ratio of two immune pathways. Platelets are among the first cells to initiate inflammation cascade. Some studies indicated that PLR may be a more accurate predictor of inflammation severity than NLR (27, 28). SII is calculated by a combination of platelets, neutrophils, and lymphocytes. A study of 290 hip fracture patients by Wang et al. found a significant association between SII and 1-year mortality (29). Monocytes and macrophages, like neutrophils, are highly phagocytic and play a key role in clearing microbes and particles marked for clearance. They are mobilized shortly after neutrophils and persist at sites of chronic inflammation and infection. They produce cytokines that regulate adaptive immune responses. Classically activated macrophages release high levels of IFN-γ, IL-6, IL-12, and TNF, showing strong proinflammatory and antibacterial effects (26). Monocytes possess the capacity to regulate and enhance inflammation, with their significant involvement in chronic inflammation potentially elucidating the superior predictive value of MLR compared to other inflammatory markers in predicting 3-year mortality.

The advantages of this study were that the baseline data was relatively complete, a large sample size and a long follow-up period were obtained. At the same time, this study still has limitations in some aspects. Firstly, the patients included in this study were all geriatric patients with hip fracture in the co-management mode, which may lead to selection bias. Secondly, blood transfusions before laboratory tests could impact inflammatory cell count results. However, obtaining transfusion history from patients transferred from other hospitals was difficult, potentially introducing bias if transfusions occurred before arrival at our hospital. Thirdly, the cause of death was not included in the study, so further analysis was not possible. In the future, high-quality prospective randomized controlled studies may be needed verify more types of indicators, so as to further explore the relationship between inflammatory indicators and postoperative mortality in elderly patients with hip fracture, and obtain more powerful evidence to guide the clinical diagnosis and management.

## 5 Conclusion

Preoperative MLR is a useful inflammatory indicator for predicting 3-year mortality in elderly hip fracture patients, but its predictive ability decreased over time.

## Data Availability

The original contributions presented in the study are included in the article/Supplementary Material, further inquiries can be directed to the corresponding authors.
