# Corrigendum: Association between admission inflammatory indicators and 3-year mortality risk in geriatric patients after hip fracture surgery: a retrospective analysis of a prospective cohort study

**DOI:** 10.3389/fsurg.2024.1506821

**Published:** 2024-11-15

**Authors:** Yimin Chen, Chao Tu, Gang Liu, Weidong Peng, Jing Zhang, Yufeng Ge, Zhelun Tan, Mingjian Bei, Feng Gao, Maoyi Tian, Minghui Yang, Xinbao Wu

**Affiliations:** ^1^ Peking University Fourth School of Clinical Medicine, Beijing, China; ^2^ Department of Orthopaedics and Traumatology, Beijing Jishuitan Hospital, Capital Medical University, Beijing, China; ^3^ National Center for Orthopaedics, Beijing, China; ^4^ School of Public Health, Harbin Medical University, Harbin, Heilongjiang, China; ^5^ The George Institute for Global Health, University of New South Wales, Sydney, NSW, Australia

**Keywords:** geriatric, hip fracture, MLR, NLR, PLR, SII, crp, mortality

A Corrigendum on Association between admission inflammatory indicators and 3-year mortality risk in geriatric patients after hip fracture surgery: a retrospective cohort study By Chen Y, Tu C, Liu G, Peng W, Zhang J, Ge Y, Tan Z, Bei M, Gao F, Tian M, Yang M and Wu X (2024). Front Surg. 11:1440990. doi: 10.3389/fsurg.2024.1440990

**Error in the Article Title**

In the published article, there was an error in the article title. Instead of “*Association between admission inflammatory indicators and 3-year mortality risk in geriatric patients after hip fracture surgery: **a retrospective cohort study***”, it should be “*Association between admission inflammatory indicators and 3-year mortality risk in geriatric patients after hip fracture surgery: **a retrospective analysis of a prospective cohort study***”.

The authors apologize for this error and state that this does not change the scientific conclusions of the article in any way. The original article has been updated.

